# The first record of ostrich feather louse (*Struthiolipeurus struthionis*) collected from farmed ostriches (*Struthio camelus*) in the United Arab Emirates

**DOI:** 10.14202/vetworld.2024.125-130

**Published:** 2024-01-18

**Authors:** Nighat Perveen, Sabir Bin Muzaffar, Mohammad Nafi Solaiman Al-Sabi, Layaly Hamdan, Adnan Aldarwich, Daniil Iliashevich, Khaja Mohteshamuddin, Olivier Andre Sparagano, Arve Lee Willingham

**Affiliations:** 1 Department of Biology, College of Science, United Arab Emirates University, Al-Ain, P.O. Box 15551, UAE; 2 Department of Veterinary Medicine, College of Agriculture and Veterinary Medicine, United Arab Emirates University, Al-Ain, P.O. Box 15551, UAE; 3 Department of Basic Medical Veterinary Sciences, Faculty of Veterinary Medicine, Jordan University of Science and Technology, P.O. Box 3030, Irbid 22110, Jordan; 4 Agricultural Sciences and Practice, Royal Agricultural University, Cirencester, GL7 6JS, UK; 5 Department of Infectious Diseases and Public Health, Jockey Club College of Veterinary Medicine and Life Sciences, City University of Hong Kong, Kowloon, Hong Kong SAR, China

**Keywords:** lice, ostrich parasites, prevalence, *Struthiolipeurus struthionis*, United Arab Emirates

## Abstract

**Background and Aim:**

Ostrich (*Struthio camelus*) farming in the United Arab Emirates (UAE) is a relatively new field of farming. Farmed ostriches are susceptible to ectoparasite infestation, which affects their production. This study was conducted to estimate the prevalence of ectoparasites on ostriches raised on a farm in Abu Dhabi Emirate.

**Materials and Methods:**

The feathers of 42 ostriches (26 females and 16 males) were collected and morphologically examined for ectoparasites. In total, 283 lice (89 males and 194 females) were collected from birds. However, there were no ticks or other ectoparasites. Lice were preserved in 1.5 mL tubes containing 70% ethanol and were later identified using taxonomic keys. The prevalence, mean intensity of infection, and mean abundance were estimated.

**Results:**

One louse species, *Struthiolipeurus struthionis* was identified. To the best of our knowledge, this is the first report of *S. struthionis* in ostriches raised in the UAE with an overall prevalence of 88%. The prevalence of lice was significantly higher in July (100%) than that in May (66.7%). Likewise, the mean intensity and abundance of lice were significantly higher in June (10.6 and 9.94, respectively) than in May (3.25 and 2.2, respectively).

**Conclusion:**

The high prevalence of lice poses a serious risk to ostrich farming by negatively affecting the health and productivity of ostriches.

## Introduction

Ectoparasite infestation of farmed animals causes global economic losses to farming industries [1, 2]. Ratite farming is a new area of animal production in the United Arab Emirates (UAE), where ostriches (*Struthio camelus*) are reared for meat, skin, and feather production. Little is known about the common ostrich ectoparasites in the UAE. Worldwide, ticks, mites, and lice are the most common ectoparasites of ostriches [2, 3]. Captive birds are more susceptible to parasitic infections compared to wild birds, which are able to leave hostile environments to avoid such health challenges [4]. Ostrich feather lice, *Struthiolipeurus struthionis* (Gervais, 1844), are external parasites of ostriches that can negatively impact their health and productivity [5]. Skin damage [6], intense pruritis, feather damage, and feather loss [5, 7] can be caused. Lice are visible on feathers close to the skin around the vent, legs, wings, and neck [6]. Eggs are deposited on feather barbs on both sides along the shaft [8, 9]. The severity of lice infestation is affected by host density, host confinement, husbandry methods, climatic conditions, and control strategies [1]. Bird ectoparasites can cause secondary infections, including bacterial, fungal, and viral infections in wounds [4]. Ostrich lice may cause problems in humans, as has been observed in pigeon. Pigeon lice may cause dermatosis in humans, as evidenced by case reports of intensely pruritic erythematous papules in human patients [10].

Other significant ostrich ectoparasites include ticks and mites. Feather mites, such as *Gabucinia bicaudata*, feed on the blood and gelatin of feather sheaths. They live in the veins in the ventral grooves of the feather shaft and can attack the skin, causing damage similar to scabies [3]. Ticks are important vectors of several viral, bacterial, and protozoan pathogens that can cause a wide range of animal and human diseases [11]. Approximately 10 species of hard ticks, including *Amblyomma gemma*, *Amblyomma lepidum*, *Amblyomma variegatum*, *Haemaphysalis punctata*, *Hyalomma albiparmatum*, *Hyalomma luscitanicum*, *Hyalomma marginatum rufipes*, *Hyalomma truncatum*, *Hyalomma* spp., and *Rhipicephalus turanicus*, were collected and identified in USA on imported ostriches [12]. Crimean–Congo hemorrhagic fever (CCHF) has also been reported in domestic animals, including ostriches [13], and among workers at an ostrich abattoir in South Africa [14]. CCHF is a tick-borne viral infection caused by the CCHF virus (CCHFV) [15], with a human case fatality rate of up to 50% [16]. CCHFV is mainly transmitted by *Hyalomma* ticks [17], which act as both reservoirs and vectors for CCHFV [18]. A high prevalence of *Hyalomma* ticks has been reported in different livestock species in UAE [19]. Therefore, continued monitoring of ostrich ectoparasites is crucial to avoid both animal and human infections.

Ostriches may thrive in a variety of habitats; however, semi-arid, open, and short-grass plains have the highest ostrich densities [20]. The Arabian ostrich was common in the Arabian Peninsula and became extinct around 1966 [21]. However, these birds were found in Syria and Saudi Arabia until the middle of the century [22]. The first commercial ostrich farm was established in South Africa in 1860 exclusively for harvesting their feathers, and subsequently expanded to other areas, including Argentina, Australia, Egypt, New Zealand, and the United States by 1913 [20]. The farms are now established in Europe, Asia, and the Middle East [23]. The first ostrich farm in the UAE was established in 1997 in Al-Ain, where the birds were imported from Namibia [24]. At present, only a few ostrich farms exist in the UAE. There is a lack of information on basic aspects of ratite production in the UAE agricultural sector, including external parasite species.

Therefore, the aims of this study were to (i) recover and identify ectoparasites infesting ostriches in the UAE based on morphological characterization, (ii) compare ectoparasite infestation between male and female ostriches, and (iii) determine the prevalence of ectoparasite species on ostriches.

## Materials and Methods

### Ethical approval

Ectoparasite collection was performed in accordance with the experimental protocol approved by the Animal Research Ethics Committee of the UAE University (ethical approval # ERA_2022_1647).

### Study period and location

The study was conducted from May to July 2022 on a private farm in the Al-Ain region of the Abu Dhabi Emirate, Abu Dhabi, UAE. The desert ecosystem of Al-Ain is characterized by high seasonal temperatures, with mean monthly temperatures varying from 17.1°C in winter to 38.1°C in summer [25]. This area is home to livestock farming, including camels, sheep, goats, and cattle. Details regarding the ostriches (males and females) sampled and lice counts from each host are provided in Table-S1 (Supplementary data).

### Ectoparasite sampling

We examined 42 randomly selected ostriches, including 16 males and 26 females. Ostriches were restrained by experienced handlers at the farm using hooding, which is the most common, safe, and reliable restraint method. A total of 283 lice (89 males and 194 females) were collected from birds. Ectoparasites were collected by visual inspection of the ostrich body and feathers [26] and placed in 50 mL plastic tubes containing 70% ethanol, with parasites from one ostrich placed in the same tube. They were then taken to the Parasitology Laboratory at the Department of Veterinary Medicine, College of Agriculture and Veterinary Medicine, UAE University, UAE, where they were kept at 20°C until further processing. Parasites in each tube were counted, and all tubes were labeled with the date of sampling, host gender (male/female ostrich), and the number of parasites.

### Morphological identification of ectoparasites

After clearing specimens in 10% KOH, the laboratory identified ectoparasites at species level based on their morphological characteristics under a dissecting Nikon SMZ1500 stereoscopic zoom microscope (Nikon, Tokyo, Japan) using taxonomic keys [27, 28]. Identification was confirmed by Prof. Bilal Dik of the Department of Parasitology, Veterinary Faculty, University of Selcuk, Konya, Turkey.

### Statistical analysis

The following parasitological indicators were determined [29, 30]:

Prevalence (%) = 100 × number of infested hosts/total number of examined hosts

Mean intensity = Number of parasites/number of infested hosts

Mean abundance = Number of parasites/total number of examined hosts

Mean intensities and abundance values were compared between male and female birds and between collection months using bootstrap t-tests, and p-values were generated using 2000 replications. Using Fisher’s exact test and 95% confidence levels using the Clopper–Pearson method, the prevalence of parasites was compared between birds based on sex. All comparisons were performed using the Quantitative Parasitology Software version 3.0 [29].

## Results

### Lice identification

**Figure-1:**
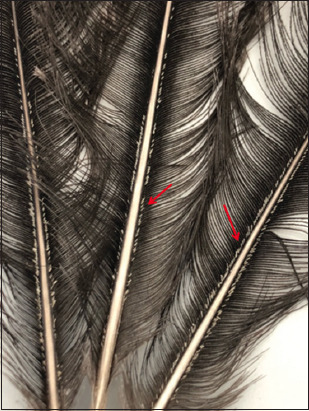
Eggs of *Struthiolipeurus struthionis* on feathers of an ostrich sampled in Al-Ain, United Arab Emirates from May to July 2022.

**Figure-2:**
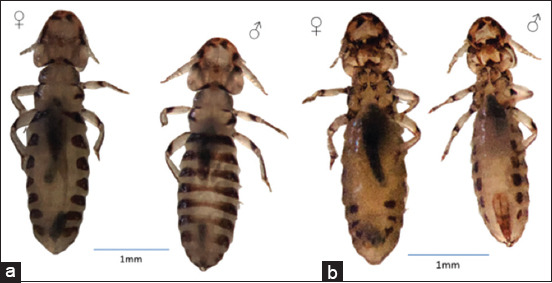
Males (♂) and females (♀) of the adult stages of *Struthiolipeurus struthionis* collected from the feathers of an ostrich on a farm in Al-Ain, United Arab Emirates from May to July 2022. Dorsal side (a), and ventral side (b).

Two hundred and eighty-three lice were collected, 89 from males and 194 from females. Eggs on feather barbs on both sides along the shaft were observed (Figure-1). Adult images of male and female lice are presented in Figure-2. No ticks or other types of ectoparasites were detected.

### Lice prevalence

The prevalence of *S. struthionis* in ostriches (at 95% confidence interval level [95% CI]) was 94% (69%–99%: 95% CI) in male birds and 85% (65%–96%: 95% CI) in female birds (Table-1). However, there was no significant difference in the prevalence of lice between male and female birds (Fisher’s exact test, p = 0.63 for pairwise comparison). The mean intensity of lice was 5.67 (3.87–8) and 9.0 (5.86–12.91) on male and female ostriches, respectively. No significant difference was observed in the mean intensity of lice between male and female ostriches (p = 0.13 for pairwise comparison, Bootstrap 2-sample t-test). Similarly, no significant difference was observed in the mean abundance of lice between male and female birds (5.31 for males and 7.62 for females; Bootstrap 2-sample t-test, p = 0.25 for pairwise comparison).

**Table-1:** Prevalence, mean intensity, and mean abundance of *Struthiolipeurus struthionis* on ostriches in Al-Ain, UAE in the period from May to July 2022.

Host sex	Numbers examined	Numbers infested	Prevalence % (95% confidence level)	Mean intensity (95% confidence level)	Mean abundance (95% confidence level)
Male	16	15	94 (69–99)	5.67 (3.87–8)	5.31 (3.44–7.50)
Female	26	22	85 (65–96)	9 (5.86–12.91)	7.62 (4.92–11.19)

UAE=United Arab Emirates

The prevalence of lice on birds in July was significantly higher (100%) than that in May (66.7%) (Fisher’s exact test, p = 0.03). However, the prevalence of lice did not differ significantly between May (66.7%) and June (93.8%) and between June and July (Fisher’s exact test, p > 0.05 for all pairwise comparisons). The mean intensity of lice was significantly higher in June (10.6; 95% CI: 7.20–14.00) than in May (3.25; 95% CI: 1.75–4.88) (Bootstrap 2-sample t-test, p = 0.001). However, there was no significant difference in the mean intensity of lice on birds between May and July and between June and July (Table-2: Bootstrap 2-sample t-test, p > 0.05 for all pairwise comparisons). The mean abundance of lice on birds was significantly higher in June (9.94; 95% CI: 6.50–13.31) than in May (2.2; 95% CI: 0.92–3.75) (Bootstrap 2-sample t-test, p = 0.001 for all pairwise comparisons). There were no differences in the mean abundance of lice on birds between May and July or between June and July (Table-2; Bootstrap 2-sample t-test, p > 0.05 for all pairwise comparisons).

**Table-2:** Monthly P, MI, and MA of *Struthiolipeurus struthionis* collected from male and female ostriches in Al-Ain, UAE in the period from May to July 2022. Numbers were presented at 95% confidence level.

Sampling months	P (95% CI)	MI (95% CI)	MA (95% CI)
May	66.7 (34.8–90)	3.25 (1.75–4.88)	2.2 (0.92–3.75)
June	93.8 (69.7–99.8)	10.6 (7.20–14.00)	9.94 (6.50–13.31)
July	100 (76.8–100)	7.0 (3.93–12.07)	7.0 (3.93–12.07)

P=Prevalence, MI=Mean intensity, MA=Mean abundance, CI=Confidence interval, UAE=United Arab Emirates

## Discussion

Ostrich farming has an extremely low carbon footprint compared to other livestock production [31], and it is relatively new in the UAE, with increasing preference among people. Only *S. struthionis* lice were detected in the examined ostriches and their prevalence was assessed over three consecutive months from May to July. Morphological analysis of the lice collected in this study confirmed the presence of *S. struthionis*. *S. struthionis* are chewing lice (*Phthiraptera*: *Ischnocera*) that do not suck blood but feed on feathers [9]. These lice species can easily disappear under feathers; therefore, it is challenging to detect them [6, 8, 9, 27]. However, lice eggs are easily visible and are deposited on feather barbs on both sides along the shaft [8, 9, 32].

This study provides the first record of *S. struthionis* in the UAE. In this study, we found a high prevalence of lice in July, as almost all birds were infested with lice. In a previous study on the effect of *S. struthionis* on leather production and leather quality in South Africa, the number of louse-infested birds increased from August (late winter) to January (summer), suggesting a seasonal increase in louse numbers [33]. To better understand the actual fluctuations in louse populations or seasonal patterns of infestation, the number of lice should be monitored throughout the year.

In this study, only one ectoparasite species, *S. struthionis*, was found on ostriches. Three species of ectoparasites, including two mite species, *G. bicaudata* and *Dermoglyphus pachycnemis*, and one louse species, *S. struthionis*, have been reported on ostriches in Egypt [2]. Microscopic examination of feathers was not conducted to identify the presence/species of mites in this study. In the present study, we detected 88% prevalence of lice (94% in males and 85% in females), whereas 3.85% prevalence was detected in ostriches in Egypt [2]. Thirty-six species of migratory birds were examined in Bursa, Turkey, with 72.2% infested and overall 58.8% infested with at least one species of chewing lice [34]. In another study conducted on domestic and wild birds, 48.23% were found to be infested with at least one louse species, with 32 louse species detected [35]. In the two northern Iranian provinces of Mazandaran and Guilan, 79 birds belonging to six bird species, including the Great Crested Grebe, Little Egret, Purple Heron, Cormorant, Black-headed Gull, and Rock Pigeon were examined with 15.2% found infested with lice [36]. Approximately 4000 lice species have been detected on birds worldwide [28].

*S. struthionis* is a chewing (biting) louse with mandibular mouth parts that ingests skin scales and scabs, causing stress to birds, reducing their weight, and making them susceptible to secondary infections and gastrointestinal disorders [5]. However, some lice species have been reported to feed on blood by gnawing through the skin and rupturing the soft quills near the base of feathers [37]. As a result, lice infestation has a negative impact on the productivity of ostriches and, ultimately, ostrich farming, resulting in economic losses. We saw a few ostriches lose their feathers in the farm. In addition, lice infestation may pose a threat to humans and animals not only because of their blood-feeding or chewing habits but also because of their ability to transmit pathogens. Most chewing lice are parasites of birds, and several of these lice are suspected vectors of avian pathogens [38], for example, aquatic and other birds are vectors of filarial nematodes [39, 40]. Ranch birds, such as emus, ostriches, and rheas, are susceptible to similar adverse effects of chewing lice [38].

Heavy tick infestations have recently been reported on different livestock species in UAE, including camels, cattle, sheep, and goats [19, 41, 42]. Ten species of ticks on imported ostriches have been previously identified including *Hyalomma* spp. [3, 12]. *Hyalomma* ticks are a vector of the CCHFV [17]. CCHFV has been reported in ostriches [13] and among workers at an ostrich abattoir in South Africa [14]. Therefore, the ostrich farming industry faces a great threat of tick-borne diseases due to the high prevalence of ticks in this area. Although no ticks were detected on ostriches in the present study, continuous surveillance is crucial to avoid future threats.

## Limitations

Our study had the following limitations: (1) We conducted the study on only one private farm; (2) handling ostriches to examine for ectoparasites required effort and labor; accordingly, the sample size was small; and (3) each month, birds were randomly selected from different pens and were investigated for only 3 months, May, June, and July; thus, the actual dynamics of louse population and seasonal patterns of infestation throughout the year could not be assessed.

## Conclusion

Ratite farming is a relatively new field of animal production in the UAE. At present, little is known about parasites, other veterinary problems, and health management of these exotic birds in the Middle East, whereas most of the information on the health and production of farmed ostriches is based on publications from South Africa. *S. struthionis* was reported to have a high prevalence in the present study, raising concerns about the potential role of lice in causing health-related changes and production losses among farmed ostriches in the UAE. Therefore, breeders and veterinarians in the UAE should have a basic knowledge of ratite ectoparasite monitoring, prevention and control, and other health management strategies to obtain maximum benefits from this new livestock industry.

## Data Availability

The supplementary data can be obtained from the corresponding author on a reasonable request.

## Authors’ Contributions

ALW and NP: Conceptualization and methodology. NP, ALW, KM, LH, AA, and DI: Sample collection. NP and SBM: Software. SBM: Validation. NP, SBM, ALW, MNSA, LH, and OAS: Formal analysis. NP: Investigations, species identification, data curation, writing-original draft preparation, visualization. NP, ALW, SBM, MNSA, OAS, LH, DI, KM, and AA: Writing-review and editing. ALW: Supervision and project administration. All authors have read, reviewed, and approved the final manuscript.
